# The Effect of Impaired Polyamine Transport on Pneumococcal Transcriptome

**DOI:** 10.3390/pathogens10101322

**Published:** 2021-10-14

**Authors:** Mary F. Nakamya, Moses B. Ayoola, Leslie A. Shack, Edwin Swiatlo, Bindu Nanduri

**Affiliations:** 1 Department of Comparative Biomedical Sciences, College of Veterinary Medicine, Mississippi State University, Starkville, MS 39762, USA; mfn35@msstate.edu (M.F.N.); mba185@msstate.edu (M.B.A.); shack@cvm.msstate.edu (L.A.S.); 2 Section of Infectious Diseases, Southeast Louisiana Veterans Health Care System, New Orleans, LA 70112, USA; edwin.swiatlo@va.gov

**Keywords:** *Streptococcus pneumoniae*, polyamine transporter, oxidative stress, nitrosative stress, transcriptome, metabolome

## Abstract

Infections due to *Streptococcus pneumoniae*, a commensal in the nasopharynx, still claim a significant number of lives worldwide. Genome plasticity, antibiotic resistance, and limited serotype coverage of the available polysaccharide-based conjugate vaccines confounds therapeutic interventions to limit the spread of this pathogen. Pathogenic mechanisms that allow successful adaption and persistence in the host could be potential innovative therapeutic targets. Polyamines are ubiquitous polycationic molecules that regulate many cellular processes. We previously reported that deletion of polyamine transport operon *potABCD*, which encodes a putrescine/spermidine transporter (Δ*potABCD*), resulted in an unencapsulated attenuated phenotype. Here, we characterize the transcriptome, metabolome, and stress responses of polyamine transport-deficient *S. pneumoniae*. Compared with the wild-type strain, the expression of genes involved in oxidative stress responses and the nucleotide sugar metabolism was reduced, while expression of genes involved in the Leloir, tagatose, and pentose phosphate pathways was higher in Δ*potABCD*. A metabolic shift towards the pentose phosphate pathway will limit the synthesis of precursors of capsule polysaccharides. Metabolomics results show reduced levels of glutathione and pyruvate in the mutant. Our results also show that the *potABCD* operon protects pneumococci against hydrogen peroxide and nitrosative stress. Our findings demonstrate the importance of polyamine transport in pneumococcal physiology that could impact in vivo fitness. Thus, polyamine transport in pneumococci represents a novel target for therapeutic interventions.

## 1. Introduction

Despite years of intensive research, infections due to *Streptococcus pneumoniae* (pneumococcus) still claim countless lives across the globe [1]. Pneumococci account for up to 15% of pneumonia cases in the USA and 27% worldwide [2]. Following colonization of the nasopharynx, pneumococci can translocate to sterile sites and cause infections such as otitis media, community-acquired pneumonia, meningitis, and septicemia [3]. Well-coordinated metabolic networks for efficient exploitation of the host micro-nutrients and immune response evasion strategies are crucial for pneumococcal pathogenesis. Serotype diversity, limited serotype coverage of the available vaccines, serotype replacement, and increase in multidrug-resistant strains confound intervention strategies that limit the spread of pneumococci [4,5,6]. A better understanding of pneumococcal physiology and survival mechanisms in the host can identify novel therapeutic targets.

Polyamines are polycationic molecules that interact with RNA, DNA, and phospholipids and modulate cellular processes such as cell division, transcription, and translation [7,8]. Putrescine, spermidine, spermine, and cadaverine are the principal cellular polyamines and their intracellular concentrations are tightly regulated by transport, biosynthesis, and degradation [8]. In pathogenic bacteria, polyamines are known to regulate virulence, biofilm formation, stress responses, in vivo fitness, and host-pathogen interactions [9]. Therefore, failure to maintain intracellular polyamine pools could impact regulatory homeostasis and interfere with in vivo survival and pathogenesis. Our previous work has shown that the polyamine transport operon *potABCD* is essential for virulence in murine models of pneumococcal infections [10]. PotD, the substrate-binding domain of the polyamine transporter is a protein antigen as it has been reported that PotD alone or in combination with other proteins elicits protection against pneumococcal colonization, pneumonia, and sepsis in mice [11,12,13]. We have shown that in a murine model of pneumonia, Δ*potABCD* is more invasive and translocates to the lungs, and is cleared faster than the wild-type strain (WT) but is more susceptible to opsonophagocytosis [14]. Uptake of Δ*potABCD* by neutrophils does not require antibody opsonization [14]. We have recently shown that deletion of the *potABCD* operon resulted in reduced intracellular concentrations of putrescine and spermidine and an unencapsulated phenotype [15,16]. Since capsular polysaccharide (CPS) is a determinant of pneumococcal virulence, reduced CPS could explain reported in vivo attenuation of Δ*potABCD*. Initial characterization of the Δ*potABCD* proteome identified altered expression of over 100 proteins, including virulence factors such as pneumolysin [10]. However, the limited proteome coverage did not allow for the identification of specific mechanisms of metabolic regulation that could explain the observed attenuated phenotype. To determine polyamine dependent metabolic regulation that is at the intersection of pneumococcal virulence, we compared the transcriptome and metabolome of *ΔpotABCD* and WT using RNA-Seq and metabolomics. Given the role of polyamines in the defense against reactive radicals, and the diversity of stress conditions encountered by pneumococci in vivo, reduced intracellular polyamine levels are expected to adversely affect stress responses that are critical for in vivo fitness. Therefore, we examined the susceptibility of *ΔpotABCD* pneumococci to oxidative and nitrosative stress. We determined the impact of impaired polyamine transport on the intracellular pH (pH_i_), reduced/oxidized glutathione (GSH/GSSG) ratio, and production of nicotinamide adenine dinucleotide phosphate (NADPH) and hydrogen peroxide (H_2_O_2_). Our results show that impaired polyamine transport renders pneumococci susceptible to stress and shifts central metabolism towards the pentose phosphate pathway (PPP), which could adversely affect the synthesis of precursors for capsular polysaccharide. Impaired stress responses and inhibition of CPS synthesis will ultimately impact in vivo survival, thus making polyamine transport an attractive anti-virulence strategy for developing novel interventions.

## 2. Results

### 2.1. Polyamine Transport Modulates Pneumococcal Gene Expression

Comparison of the transcriptome profiles of TIGR4 and Δ*potABCD* identified pneumococcal pathways responsive to impaired polyamine transport. Using a ≥ 1.3-fold change cut off, we identified a total of 1333 differentially expressed genes (DEGs) whose expression varied significantly between the WT and the deletion strain. Expression of 651 and 682 genes was downregulated and upregulated in Δ*potABCD*, respectively (Table 1, Table 2, Table 3 and Table S2). However, when a more stringent cut off (≥2-fold change) was used, 273 DEGs were identified, representing 11.5% of TIGR4 genome, and were used for further analysis where expression of 81 and 192 genes was downregulated and upregulated in Δ*potABCD*, respectively (Table 1, Table 2, Table 3 and Table S2). Gene Ontology analysis of the DEGs identified significant enrichment of five categories which included: phosphotransferase systems (PTS), galactose metabolism, ABC transporters, amino and nucleotide sugars, and fructose and mannose metabolism. Major biological functions and pathways represented by the DEGs (≥2-fold change) and metabolites are discussed in the following sections and shown in Table 1, Table 2, Table 3, and Table 4, respectively.

### 2.2. Polyamine Transporter and Pneumococcal Stress Responses

Polyamines protect cells against reactive oxygen species (ROS) by directly scavenging reactive free radicals or via the regulation of stress response genes [8,17]. Therefore, reduced levels of intracellular polyamines reported in Δ*potABCD* could alter the redox status and render Δ*potABCD* susceptible to oxidative stress. Downregulation of genes that encode *treR*, a scavenger of H_2_O_2_, molecular chaperones and their regulator, *hrcA* (Table 1), indicate impaired redox and repair systems which could compromise in vivo fitness of Δ*potABCD* [18,19]. Reduced expression of genes which encode several tRNAs (Table S2) could impact protein synthesis, and thus cellular adaptation to stress and maintenance of redox homeostasis [20]. Increased expression of genes that encode regulators implicated in pneumococcal stress responses, the arginine deiminase system (ADS), and glutamine transporters could be in response to higher oxidative stress in the mutant [21,22] (Table 1). Elevated glutamine influx and ADS could meet the increased demand for energy (ATP) and restore the buffering capacity (ammonia). Increased expression of ABC transporters for the import of iron, manganese, and phosphate (Table 1) could result in cationic imbalance and negatively impact cellular functions and redox homeostasis [23]. These results show that deficiency of the polyamine transporter impairs pneumococcal stress systems, renders the mutant susceptible to oxidative stress, and triggers the expression of polyamine independent redox systems probably to combat the stress.

### 2.3. Galactose Utilization and the Pentose Phosphate Pathway (PPP)

The signature of oxidative stress in the mutant was further revealed by the observed metabolic shift towards the PPP in the mutant which is usually in response to oxidative stress [24]. PPP generates NADH/NADPH which are cofactors for antioxidant enzymes such as, thioredoxin reductase [25]. Expression of genes that encode enzymes of the Leloir pathway involved in galactose catabolism and generation of UDP-galactose (UDP-Gal) [26] was upregulated in the mutant (Table 2). Expression of *lacF-2* and the *lacDCBA* operon involved in the import and interconversion of galactose via the tagatose pathway to fructose 6-phosphate and glyceraldehyde 3-phosphate (G3P) [27,28,29] was upregulated (Table 2). Upregulation of Leloir and tagatose pathways could be in response to the high demand for PPP precursors in the mutant. Increased PPP and G3P may contribute to the upregulation of *tktC* and *tktN* which encode a transketolase, the enzyme that catalyzes the interconversion of sugar-phosphates in the PPP pathway [30] (Table 2). There was an increase in the expression of genes that encode enzymes that degrade fucose (Table 2). Fucose degradation by triosephosphate isomerase yields G3P, an intermediate of glycolysis and PPP [25]. Increased expression of a putative PTS system involved in the import and interconversion of L-ascorbate to xylulose 5-phosphate, an intermediate of PPP (Table 2) further suggests increased activity of the PPP. Genes involved in ascorbate utilization are co-transcribed upstream with a transcriptional regulator (*bglG*) whose expression was upregulated in Δ*potABCD* (Table 2). Carbon shunt via the PPP could be to meet the demand for NADPH due to increased oxidative stress in Δ*potABCD*.

### 2.4. Glycolysis and Production of Precursors for the Pneumococcal Capsule

During stress, organisms modulate their gene expression to limit energy-consuming processes to preserve energy for redox systems [31,32]. Gene expression profile of *ΔpotABCD* indicates a limited flow of carbohydrates through the main glycolytic pathway. Expression of a sucrose operon regulator and genes involved in sucrose uptake was reduced (Table 3). Expression of *malP*, which encodes an enzyme that breaks down glycogen to glucose 1-phosphate and the *malXCD* operon, which encodes a maltose/maltodextrin transporter, was downregulated (Table 3). Reduced influx of sucrose, maltose, and reduced breakdown of glucose in glycolysis will deplete glycolytic intermediates in Δ*potABCD*. UDP-N-acetylglucosamine (UDP-GlcNAc) is a precursor for three sugars in pneumococcal CPS repeat unit (UDP-N-acetylmannosamine (UDP-ManNAc), UDP-N-acetylgalactosamine (UDP-GalNAc), and UDP-N-acetylfucosamine (UDP-FucNAc) [33,34]. UDP-GlcNAc levels in the mutant are expected to be low due to elevated levels of *nagA* and *nagB* involved in its breakdown to fructose 6-phosphate [35] (Table 3); consequently, there will be reduced levels of precursors for CPS synthesis. Impaired structural component production was further apparent with the reduced expression of genes involved in the transport of N-acetylgalactosamine, a precursor for the peptidoglycan layer (PG), [36] (Table 3).

Moreover, the *pyr* operon involved in the de novo synthesis of pyrimidine nucleotides and *pyrR*, the regulator of this operon, were repressed (Table 3). Repression of genes from the pyrimidine biosynthesis pathway will reduce UTP, a precursor for UDP required for the activation of UDP-sugars for CPS synthesis [37]. Expression of *asd* and *dap* involved in the synthesis of lysine, a constituent of the pneumococcal PG, was reduced (Table 3). The overall effect of the above gene expression changes is reduced carbon flow through glycolysis, possibly due to higher oxidative stress in Δ*potABCD* which mandates higher PPP activity, and this will impact the production of precursors for CPS repeat unit sugars and the PG layer in the mutant.

To validate RNA-Seq results, we measured the expression of selected genes using qRT-PCR Genes that code for *tktC, tktN*, the ascorbate regulator (*bglG*), and the choline-binding protein *pcpA* were upregulated (Table 5), which is consistent with RNA-Seq results. Although not differentially expressed in the RNA-Seq results, polyamine synthesis genes *speE* and *speA* were upregulated in our qRT-PCR results. Despite this upregulation of synthesis, measurement of intracellular polyamines by metabolomics revealed reduced levels of all the major polyamines in Δ*potABCD* [16], which is consistent with our RNA-Seq analysis.

### 2.5. Redox State and Regulation of Intracellular pH in S. pneumoniae

**Figure 1:**
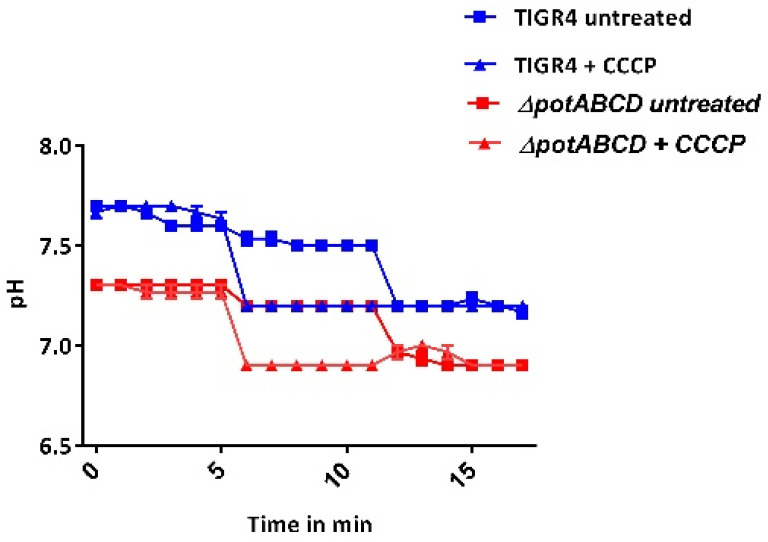
Intracellular pH of *S. pneumoniae* TIGR4 and Δ*potABCD*. Three replicates of TIGR4 and Δ*potABCD* were loaded with 5 mM of pH sensitive fluorescence dye BCECF-AM, washed with PBS, re-energized with 10% glucose, and baseline fluorescence readings were established in the first 5 min. Controls were supplemented with 10 µM CCCP as a protonophore (triangles), and fluorescence of CCCP-treated controls and untreated samples (squares) was measured for an additional 5 min. The pH_i_ of untreated TIGR4 (blue squares) and Δ*potABCD* (red squares) is 7.5 and 7.2, respectively. Nigericin (20 µM) was added to both treated and untreated samples to dissipate transmembrane gradients over the last 5 min. Graphs represent the mean of three independent experiments.

Bacteria adapt to changing microenvironments by modifying metabolite levels to maintain redox homoeostasis. To gain further insight into the role of polyamine transport on the pneumococcal stress signature observed at the transcriptome level, intracellular levels of NADPH, endogenous H_2_O_2_, pH_i_, and the GSH/GSSG ratio between WT and Δ*potABCD* were compared. Our results show that the pH_i_ of TIGR4 (7.5) and that of the mutant (7.2) were both within physiological range (Figure 1). There was no significant difference in the amount of NADPH produced by Δ*potABCD* (3.8 ± 0.4 µg/mg) compared with the WT (4.0 ± 0.6 µg/mg). We observed no significant difference in the amount of H_2_O_2_ generated by Δ*potABCD* (1 mM ± 0.05) and WT (1 mM ± 0.01). However, there was a significant difference in the GSH/GSSG ratio between the WT (1.3 ± 0.1) and Δ*potABCD* (1.7 ± 0.1, *p* ≤ 0.05). A higher GSH/GSSG ratio indicates increased GSH production, for which one stimulus is oxidative stress. These results show that intracellular levels of NADPH, H_2_O_2_, and pH_i_ are not dependent on polyamine transport.

### 2.6. Metabolic Profile of Polyamine Transport-Deficient S. pneumoniae

Our metabolomics analysis identified significant differences in the levels of several metabolites in response to *potABCD* deletion (Table 4). Levels of N-acetylglucosamine (GlcNAc) and pyruvate were reduced, which could impact CPS production in Δ*potABCD*, consistent with the gene expressions. Increased activity of the PPP was evident by the higher levels of sedoheptulose 1, 7-bisphosphate, a precursor for erythrose 4-phosphate and ribose 5-phosphate. RNA-Seq analysis indicated an increased Leloir pathway, which was confirmed by high levels of UDP-glucose, a precursor for glucose 1-phosphate, an intermediate of glucose 6-phosphate that can be channeled to PPP. The metabolome also showed reduced concentration of trehalose 6-phosphate, which could impact oxidative stress responses in Δ*potABCD*. The concentration of glutathione was higher, which could be in response to oxidative stress in Δ*potABCD*. In summary, our metabolomics results suggest oxidative stress in Δ*potABCD*, which is concordant with the RNA-Seq results.

### 2.7. PotABCD Is Required for Pneumococcal Hydrogen Peroxide and Nitrosative Stress Response

**Figure 2:**
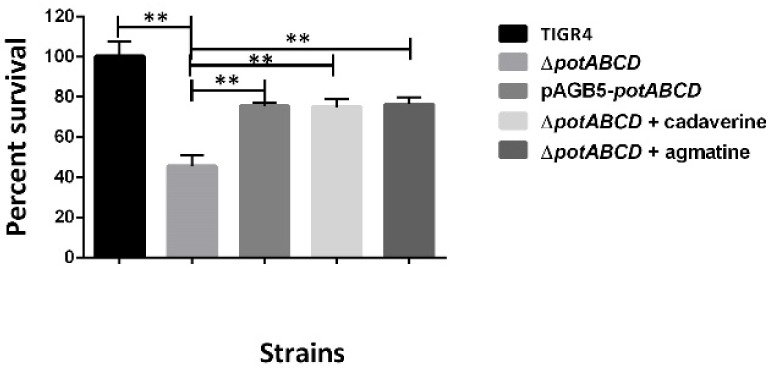
Hydrogen peroxide susceptibility of *S. pneumoniae* TIGR4, Δ*potABCD* and pABG5-*potABCD.* The graph shows Δ*potABCD* sensitivity to 2.5 mM H_2_O_2_ at 15 min post exposure. Additionally, included is Δ*potABCD* supplemented with ¼ MIC cadaverine or agmatine. The results represent an average of three independent experiments. Percentage survival relative to the WT is shown as a bar with the standard error of the mean, with ** representing *p* ≤ 0.01, determined by Student’s *t*-test.

Host defense against bacterial pathogens includes generation of ROS such as superoxide anions (O_2_^−^), H_2_O_2_, and hydroxyl radicals (OH∙), as well as reactive nitrogen species (RNS). To further corroborate the stress signature observed at the transcriptome and metabolome levels, we compared the susceptibility of TIGR4 and Δ*potABCD* to unstable oxygen and nitrogen radicals. Our results show that Δ*potABCD* is more susceptible to hydrogen peroxide stress compared with the WT. When cultured in the presence of low concentrations of exogenous hydrogen peroxide (0.5, 0.75 and 1 mM), there was no significant effect on the growth of WT and Δ*potABCD* at 15- and 30 min post exposure (data not shown). However, in the presence of 2.5 mM H_2_O_2_, viability of Δ*potABCD* was reduced by 54% relative to WT at 15 min post exposure. Survival in the presence of 2.5 mM H_2_O_2_ was restored to ~75% relative to WT in the complement pABG5-*potABCD* strain or supplementation with ¼MIC cadaverine (2.5 mM) or agmatine (20 mM) (Figure 2). Polyamine supplementation was carried out with Δ*potABCD* cultured in CDM, which lacks polyamines.

When we measured the impact of the deletion of *potABCD* on H_2_O_2_ production, we observed no significant difference in the amount of H_2_O_2_ generated by Δ*potABCD* compared with the WT strain (data not shown). This result indicates that it is not higher endogenous H_2_O_2_ production which rendered Δ*potABCD* more susceptible to exogenous H_2_O_2_.

**Figure 3:**
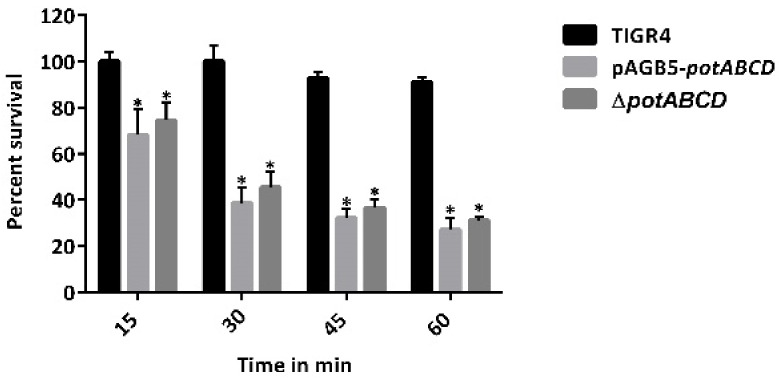
*S*-nitrosoglutathione susceptibility assay of *S. pneumoniae* TIGR4, Δ*potABCD* and pABG5-*potABCD*. The graph shows bacterial sensitivity to 2.5 mM GSNO at 15–60 min post exposure. The results represent an average of three independent experiments. Percentage survival relative to the untreated control is shown as a bar with the standard error of the mean, with * representing *p* ≤ 0.01 based on Student’s *t*-test.

Compared with WT, Δ*potABCD* was significantly more susceptible to S-Nitrosoglutathione (GSNO) that generates RNS. Exposure to GSNO at a concentration of 2.5 mM resulted in a significant reduction in the percentage survival of Δ*potABCD* that ranged between 32% at 15 min and 73% at 60 min post exposure, compared with the WT whose survival was not affected at 15 and 30 min with only 10% reduction in viability 60 min post exposure. There was no significant difference in the survival of the complement pABG5-*potABCD* strain or Δ*potABCD* supplemented with polyamines in the presence of GSNO (Figure 3). These results suggest that Δ*potABCD* is more susceptible to H_2_O_2_ and nitrosative stress than WT. Data with pABG5-*potABCD* complement (Figure 3) or polyamine supplementation (data not shown) indicate that polyamine transport is not the primary response to RNS.

## 3. Discussion

**Figure 4:**
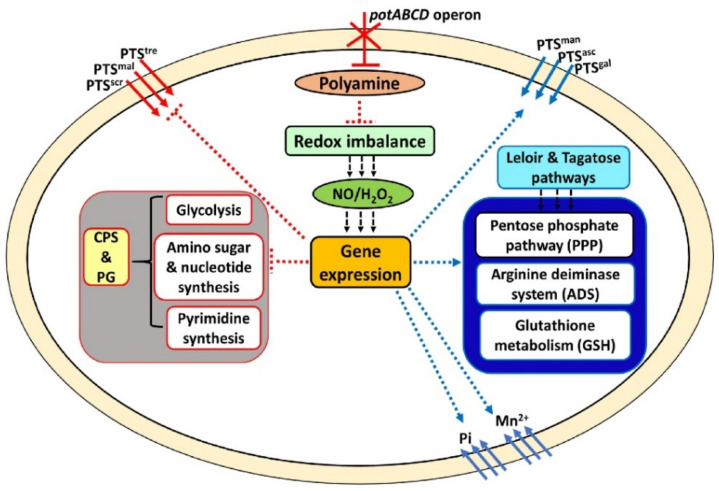
Intersection between polyamine metabolism, stress responses, carbohydrate metabolism, and CPS/PG production in *S. pneumoniae.* Multi-step reactions are shown as dotted arrows, (blue represents upregulated while red represents downregulated reactions). Deletion of the polyamine transporter results in reduced intracellular polyamine levels (red oval), impairing the redox system (light green rectangle), rendering the pneumococci susceptible to NO and H_2_O_2_ stress (dark green oval). The stressful conditions alter gene regulation (orange rectangle) to limit processes (grey square; glycolysis, pyrimidine, and amino sugar nucleotide synthesis) that yield precursors for CPS and PG synthesis (yellow vertical rectangle). A shift to the PPP could be to limit production of precursors for synthesis of CPS and PG, to save energy for redox homeostasis (dark blue vertical rectangle; ADS, PPP, and GSH metabolism) and cation inflow. Influx of carbohydrates via phosphotransferase systems (PTS) that promote synthesis of glycolysis is inhibited in favor of inflow of carbohydrates which favor the Leloir pathway and tagatose pathways (light blue rectangle), probably to provide PPP intermediates.

Findings in this study show that a deficiency in polyamine transport impairs pneumococcal stress responses and shifts central metabolism towards the PPP. Limited flow of carbohydrates through glycolysis will impair production of precursors for CPS and PG synthesis (Figure 4). In response to polyamine transport deficiency, Δ*potABCD* triggered expression of known stress response systems to restore the redox status. Acquisition of Pi and manganese is essential for normal growth, virulence, and oxidative stress responses in many pathogenic bacteria [18,23,38]. The PPP is often upregulated as a quick cellular response to meet cellular demand for NADPH to combat oxidative stress [25]. The ADS produces ammonia (NH_3_) which protects cells against acid stress and a molecule of ATP for basal cellular functions during stress [39,40]. Reduced glutathione and glutathione metabolism play a significant role during oxidative stress in many organisms [41,42,43]. Therefore, increased expression of genes involved in cation influx, PPP, the ADS, and a high GSH/GSSG ratio could be in response to stress in Δ*potABCD*. [18,44]. Upregulation of the ADS could explain the observation that Δ*potABCD* can maintain pH_i_ in the physiological range. The polyamine modulon represents genes whose expression is regulated by polyamines and is well described in *Escherichia coli*. Polyamine modulon includes genes involved in cell proliferation, biofilm formation, and detoxification of ROS [45]. Our results show impaired polyamine transport altered the expression of several regulators, and thus indirectly altering expression of genes they control. We observed that some polyamine transporter responsive genes are also part of the CcpA regulatory network, for example genes involved in stress responses, amino acid synthesis, and carbohydrate metabolism [46,47]. These results suggest that the intersection of polyamine metabolism and CcpA regulatory network is important for in vivo fitness. Future studies to identify mechanisms of co-regulation of polyamine metabolism and other transcriptional regulators that impact pneumococcal virulence are warranted.

Upregulation of the Leloir and the tagatose pathways that produce PPP intermediates and transketolase, a key enzyme in PPP, further suggest decreased carbon flow through glycolysis, supported by decreased pyruvate levels in Δ*potABCD*. Decreased glycolysis could result in reduced acetyl-CoA, a precursor for UDP-GlcNAc, and a donor for the three N-acetylated sugars at the intersection of CPS and PG repeat unit biosynthesis [48,49]. Upregulation of genes involved in the Leloir pathway in Δ*potABCD* is possibly due to the regulation of this pathway by arginine decarboxylase (*speA*). Our earlier report showed downregulation of genes of the Leloir pathway in Δ*speA*, implicating a role for polyamine synthesis in the regulation of this pathway [15], by mechanisms that are yet to be described. The PPP generates ribulose 5-phosphate which can either be used for nucleotide synthesis or converted to sedoheptulose 1, 7-bisphosphate, a precursor for erythrose 4-phosphate and G3P. High levels of sedoheptulose 1, 7-bisphosphate suggest that Δ*potABCD* promotes PPP activity possibly to increase production of NADPH levels required by redox systems. Downregulation of the *pyr* operon, responsible for the interconversion of uracil and uridine monophosphate (UMP), could impact UDP production necessary for UDP-sugar repeat unit synthesis and further impair CPS biosynthesis, and explain the unencapsulated phenotype that we described for Δ*potABCD* strain [16]. Although expression of genes of the CPS locus were not altered in our RNA-Seq results, we reported earlier that expression of capsular polysaccharide biosynthesis protein (Cps4F) was downregulated at the protein level [10]. Although, *murB* a gene involved in PG synthesis is co-transcribed with the *potABCD* operon in *S. pneumoniae* and *S. suis* [50,51], it was not identified in our transcriptome analysis. Instead, we observed reduced expression of *asd* and *dap* involved in the biosynthesis of lysine, a component of the PG repeat unit, in pneumococci. In *S. pneumoniae* regulation of PG by the polyamine transporter could be via a different mechanism that is indirect from *murB* as it was shown in *S. suis* [50]. Results from this study and our previous reports [15,16] suggest that polyamine mediated CPS and PG regulation is dependent on both polyamine transport and synthesis. Reduction in CPS precursors could explain the unencapsulated phenotype [16] and attenuation of Δ*potABCD* in murine models of colonization, pneumonia, and sepsis [10].

Hydrogen peroxide produced by the pneumococcus is essential for its pathogenesis and protection against other common respiratory tract inhabitants. H_2_O_2_ is cytotoxic to host cells, causes apoptosis in respiratory epithelial cells, and promotes colonization of the upper respiratory tract [52]. Therefore, pneumococci must adapt to survive the high levels of H_2_O_2_ produced via the pyruvate oxidase system. Despite a high GSH/GSSG ratio, maintenance of NADPH levels and upregulation of several genes involved in oxidative stress responses, Δ*potABCD* was more susceptible to exogenously added hydrogen peroxide and GSNO compared with the WT. Susceptibility of TIGR4 and Δ*potABCD* to a concentration of either 0.5, 0.75 or 1 mM H_2_O_2_ was comparable, which indicates inherent protective mechanisms at the known concentrations of endogenous H_2_O_2_ (1 mM) produced by pneumococci [53,54]. In addition, intracellular H_2_O_2_ levels in Δ*potABCD* and TIGR4 were comparable, suggesting that the increased susceptibility of Δ*potABCD* to H_2_O_2_ cannot be attributed to increased endogenous H_2_O_2_. These results suggest that polyamines could be essential for the regulation of up/down stream functions of pneumococcal stress responses. These results are consistent with the known roles of polyamines in other bacterial pathogens [8]. Putrescine and spermidine protect cells from ROS by increasing expression of genes that code for free radical scavengers [55,56]. Polyamine synthesis [57] or transport genes are upregulated [9] in response to H_2_O_2_ stress. We reported that *speA*, a gene which encodes an arginine decarboxylase, regulates pneumococcal nitrosative, H_2_O_2_, and superoxide stress responses [58]. Cadaverine protects *Salmonella typhimurium* and *E. coli* against nitrosative and acid stress [59,60]. Exogenous supplementation with either agmatine or cadaverine restores viability in Δ*potABCD* treated with H_2_O_2_ but not GSNO, although there is no annotated lysine: cadaverine antiporter in TIGR4 genome. Our results suggest that the protective ability of exogenous polyamines depends on the type of stress and concentration of the polyamine used in the experiment. Results of susceptibility assays with the complement strain also showed differences in restoring viability depending on the stress being investigated, i.e., providing transport function resulted in a significant increase in resistance only with hydrogen peroxide and not *S*-nitrosoglutathione. These results indicate that polyamine transport has a direct involvement in adaptation to H_2_O_2_ stress while it appears to have indirect effects in pneumococcal response to GSNO.

This study shows that the *potABCD* operon is involved in hydrogen peroxide and nitrosative stress responses in pneumococci. Polyamines contribute to cellular homeostasis by scavenging reactive radicals or balance pH_i_ through the consumption of a proton during their synthesis via the decarboxylation of amino acids [57,61]. Therefore, reduced intracellular polyamine levels reported earlier [16], and decreased expression of some genes involved in redox balance in the current study, could render Δ*potABCD* susceptible to stress. In addition, polyamines regulate transcription of several genes including those involved in oxidative stress responses. Downregulation of expression of *treR* which impacts the levels of trehalose, a scavenger of ROS, and the impaired DNA repair system could render the mutant sensitive to stress. The damage caused by H_2_O_2_ can be amplified via the Fenton reaction with the generation of hydroxyl radicals, the primary cause of damage to biomolecules such as DNA [62]. Therefore, increased expression of the iron transporter could aggravate the effects of oxidative stress and impair pneumococcal survival in vivo as it moves through different host niches.

In summary, deletion of polyamine transport appears to increase intracellular oxidative and nitrosative stress that results in changes in the central metabolism which favor stress responses over the synthesis of CPS and PG. Our previous results show reduced intracellular levels of putrescine and spermidine and loss of CPS in Δ*potABCD*. This study demonstrates that despite upregulation of metabolic pathways that are critical for stress responses, Δ*potABCD* is relatively more susceptible to H_2_O_2_ and GSNO, which could also contribute to reduced ability to survive host oxidative and nitrosative stress. The extent and nature of increased intracellular stress due to altered polyamine homeostasis in Δ*potABCD* warrant further investigation. The intersection of polyamine homeostasis and stress responses that modulate capsule production is an avenue for the discovery of novel vaccine and therapeutic targets against this deadly pathogen. Belenky and colleagues demonstrated that bactericidal antibiotics kill bacteria by inducing oxidative stress [63]. It has also been shown that alterations in cellular carbon flux impact antibiotic susceptibility in multiple bacterial species [64]. Therefore, altered carbon flow and increased susceptibility of the polyamine-deficient mutant suggest synergistic action to bacterial killing of bactericidal antibiotics, which warrants further investigation. Polyamine transport systems are conserved across pneumococcal serotypes and are a promising therapeutic avenue due to their immunogenic potential [11,13]. Future studies focusing on uncovering the interconnected network of pneumococcal polyamine redox homeostasis, CPS, and PG synthesis will contribute towards the deconvolution of the complex regulatory networks that impact stress responses and pneumococcal adaptation in vivo.

## 4. Materials and Methods

### 4.1. Bacterial Strains and Growth Conditions

*S. pneumoniae* serotype 4 strain TIGR4 [65], Δ*potABCD* [10] and the complement strain (pABG5-*potABCD*) [16] were used in this study. All strains were grown in either chemically defined medium (CDM) [66] or Todd-Hewitt broth supplemented with 0.5% yeast extract (THY) or on 5% sheep blood agar plates (BAP) in 5% CO_2_ at 37 °C. All assays were performed in triplicate in three independent experiments.

### 4.2. RNA Sequencing

Total RNA was isolated and purified from mid-log phase cultures (OD600 0.4–0.45) of TIGR4 and Δ*potABCD* (*n* = 4) grown in THY (a complete medium that mimics nutrients in the host milieu) using the RNeasy ^®^ Mini Kit (Qiagen, Valencia, CA, USA). RNA quality was checked with an Agilent 2100 Bioanalyzer (Agilent Technologies, Santa Clara, CA, USA). RNA-Seq analysis was performed as described earlier [16]. Briefly, libraries for RNA-Seq were prepared with the KAPA RNA Hyper Kit with RiboErase (KAPA Biosystem, Wilmington, MA, USA) with 5 µg RNA as input. The concentration and quality of libraries were determined by the Qubit ds DNA HS Assay Kit (Life Technologies, Carlsbad, CA, USA) and Agilent Tapestation (Agilent Technologies, Santa Clara, CA, USA). Sequencing was done on Illumina Hiseq 3000, the quality of the data was checked with Illumina SAV and de-multiplexing was performed with Illumina Bcl2fastq2 v 2.17. Removal of failed reads, mapping of the short sequence reads to *S. pneumoniae* TIGR4 reference genome (available at https://www.ncbi.nlm.nih.gov/assembly/GCF_000006885.1) (accessed on 11 October 2021) and identification of differentially expressed genes were performed with CLC Genomic Workbench 20.0.3 (Qiagen, Valencia, CA, USA).

Paired end reads of both WT and Δ*potABCD* were mapped to the TIGR4 genome using a CLC proprietary read mapper; read counts were estimated by EM estimation algorithm [67] and differentially expressed genes (DEGs) were identified based on the fold change generated by the edgeR algorithm. Changes in gene expression with a fold change of ± 1.3 at a false discovery rate (FDR, includes multiple testing correction) of ≤0.05 were considered significant. Functions and pathways represented by DEGs were identified, utilizing multiple bioinformatics resources such as MetaCyc [68], Gene Ontology [69], KEGG [70], UniProt [71], and STRING [72]. RNA-Seq raw data and metadata are available at NCBI GEO with the accession number PRJNA722029. https://www.ncbi.nlm.nih.gov/sra/PRJNA722029 (accessed on 11 October 2021).

### 4.3. Quantitative Real Time PCR

To validate RNA-Seq results, we measured the expression of selected genes by quantitative reverse transcription PCR (qRT-PCR). The primers used for qRT-PCR are listed in the Supplementary Materials (Table S1). All primers were validated by performing a melt curve analysis with SYBR Green (Thermo Fisher Scientific Waltham, MA, USA). In brief, total RNA was purified from mid-log phase cultures (OD600 0.4–0.45) of TIGR4 and Δ*potABCD* grown in THY (*n* = 3). Purified RNA (7.5 ng/reaction) was reverse-transcribed into cDNA and PCR was performed using the SuperScript III Platinum SYBR Green One-Step qRT-PCR Kit (Thermo Fisher Scientific, Waltham, MA, USA) as previously described [73]. Relative quantification of gene expression was determined by the Stratagene Mx3005P qPCR System (Agilent, Santa Clara, CA, USA). Expression of selected genes was normalized to the expression of *gapdh* and fold changes determined by the comparative C_T_ method.

### 4.4. Measurement of Intracellular pH

The intracellular pH (pH_i_) was determined based on the method described by Clementi and colleagues [74] with slight modifications. Briefly, mid-log phase cultures (OD600 0.4–0.45) of TIGR4 and Δ*potABCD* grown in THY (*n* = 33) were collected by centrifugation, washed, and suspended in phosphate-buffered saline (PBS). Cells (10^8^ CFU/mL) were loaded with 5 mM BCECF/AM dye (Millipore-Sigma, St. Louis, MO, USA) and incubated for 30 min at 30 °C in the dark. Cells were then pelleted, washed, and reenergized with 10 mM glucose in PBS. To obtain the in vivo calibration curve for each strain, 400 µL of energized cells were pelleted and suspended in potassium buffers ranging from pH 6.5 to 8.0. Nigericin (1 mM) (Thermo Fisher Scientific, Waltham, MA, USA) was added to the cells (to equilibrate the pH_i_ of the cells to the pH of the surrounding buffer) and incubated at 37 °C for 5 min. Fluorescence was then measured by a Synergy H1 plate reader (BioTek, Winooski, VT, USA), and a calibration curve was obtained by plotting fluorescence against the pH of the buffers. To measure the pH_i_ of individual samples, 200 µL (10^8^ CFU/mL) of the loaded and energized cells was added to the wells of a 96-well plate in duplicate and fluorescence detected using a plate reader for 5 min. A total of 10 µM carbonyl cyanide 3-chlorophenylhydrazone (CCCP) was added to one well (to serve as control) and the reading was taken after 5 min. CCCP is a protonophore that uncouples proton motive force and causes a rapid decrease in pH_i_ (Millipore-Sigma, St. Louis, MO, USA). Nigericin was added to both CCCP-treated control and untreated sample (to a final concentration of 1 mM) to equilibrate the pH_i_ to the pH of the buffer and fluorescence was read for an additional 5 min. Fluorescence was calculated and the pH_i_ interpolated from the calibration curve.

### 4.5. Measurement of Intracellular Nicotinamide Adenine Dinucleotide Phosphate (NADPH)

The intracellular concentration of NADPH was determined using the NADP/NADPH Assay Kit (Abcam, Cambridge, MA, USA). Mid-log phase cultures (OD600 0.4–0.45) of TIGR4 and Δ*potABCD* grown in THY (*n* = 33) were harvested at 5000× *g* for 10 min at 4 °C, suspended in PBS and transferred to beadbeater tubes (MP Biomedicals, Irvine, CA, USA). Cell suspensions were lysed with a FastPrep-24 ™ Classic benchtop homogenizer (MP Biomedicals, Irvine, CA, USA) and centrifuged at 6000× *g* for 5 min at 4 °C. The cells were processed according to the manufacturer’s instructions. NADPH concentrations were determined with a SpectraMax ^®^ M5 multi-mode microplate reader (Molecular Devices, Sunnyvale, CA, USA). The concentration of the protein extracts was determined with the Pierce BCA Protein Assay Kit (Thermo Fisher Scientific, Waltham, MA, USA) and used to normalize NADPH concentrations.

### 4.6. Measurement of Intracellular Glutathione

The ratios of reduced (GSH) to oxidized (GSSG) intracellular glutathione concentrations were determined using the GSH/GSSG-Glo ™ Assay Kit (Promega, Madison, WI, USA). Cells from mid-log phase cultures (OD600 0.4–0.45) of TIGR4 and Δ*potABCD* grown in THY (*n* = 33) were processed, and protein concentration was determined as with the NADPH quantification above. Luminescence was measured with a Cytation ™ 5 cell imaging multi-mode reader (BioTek, Winooski, VT, USA) and used to calculate glutathione concentrations. The concentration of the protein extracts was determined with the Pierce BCA Protein Assay Kit (Thermo Fisher Scientific, Waltham, MA, USA) and used to normalize glutathione concentrations. GSH/GSSG (reduced/oxidized glutathione) ratios were calculated from the normalized glutathione concentrations according to the manufacturer’s instructions.

### 4.7. UPLC-HRMS Untargeted Metabolomics

Approximately 10^9^ CFU/mL of cells from mid-log phase cultures (OD = 0.40–0.45) of TIGR4 and Δ*potABCD* grown in THY (*n* = 35) were transferred onto a 0.2 µm Whatman polycarbonate membrane by vacuum filtration. The membranes were snap-frozen in liquid nitrogen and stored at −80 °C. Metabolites were extracted from bacteria on the membranes with extraction solvent (40:40:20 methanol, acetonitrile, and water with 0.1% formic acid) at 4 °C. The extracts were transferred to 2.0 mL tubes, centrifuged for 5 min at 16,100× *g* at 4 °C and the supernatant transferred to new 2.0 mL tubes. Tubes containing ~1.7 mL of the total supernatant were dried under a stream of N_2_, and solid residue was suspended in 300 µL of sterile water and transferred to autosampler vials for mass spectrometric analysis. A 10 µL aliquot was injected through a Synergi 2.5-micron reverse-phase Hydro-RP 100, 100 × 2.00 mM LC column (Phenomenex, Torrance, CA, USA) kept at 25 °C. The eluent was introduced into the MS via an electrospray ionization source conjoined to an ExactiveTM Plus Orbitrap Mass Spectrometer (Thermo Scientific, Waltham, MA, USA) through a 0.1 mm internal diameter fused silica capillary tube. The mass spectrometer was run in full scan mode with negative ionization mode with a window from 85 to 1000 *m*/*z* with a method adapted from [75]. Samples were run with a spray voltage of 3 kV. The nitrogen sheath gas was set to a flow rate of 10 psi with a capillary temperature of 320 °C. Automatic gain control target was set to 3e6. The samples were analyzed with a resolution of 140,000 and a scan window of 85–800 *m*/*z* from 0 to 9 min and 110–1000 *m*/*z* from 9 to 25 min. Files generated by Xcalibur (RAW) were converted to the open source mzML format [69] via the open source msconvert software as part of the ProteoWizard package [69]. Maven (mzroll) software, (Princeton University) was used to automatically correct the total ion chromatograms based on the retention times for each sample [76,77]. Metabolites were manually identified and integrated using known masses (±5 ppm mass tolerance) and retention times (1 ≤ 1.5 min). Unknown peaks were automatically selected via Maven’s automated peak detection algorithms. A database of 275 metabolites verified using exact m/z and known retention times, expanded from the original database [75] was used. The statistical analysis on metabolite peak intensity post CFU normalization was done by MetaboAnalyst 4.0 [78]. Quantile normalization which is highly efficient in normalizing metabolite variations from mass spectrometry [79] was used to normalize the data. Significant differences in metabolite peak intensity between Δ*potABCD* and TIGR4 were identified by a *t*-test at an adjusted FDR of ≤0.05.

### 4.8. Hydrogen Peroxide Production

H_2_O_2_ generated from mid-log phase cultures (OD = 0.40-0.45) of (10 mL) of TIGR4 and Δ*potABCD* (*n* = 33) was compared using a quantitative peroxide assay (Pierce, Thermo Fisher Scientific Waltham, MA, USA). Briefly, 1 mL of bacterial culture (10^8^ CFU/mL) grown in THY was centrifuged at 4 °C for 2 min at 10,000× *g* and the supernatant filtered with a 0.22 µm filter. The concentration of H_2_O_2_ was measured in the filtrate following the manufacturer’s instructions.

### 4.9. Hydrogen Peroxide Susceptibility

Mid-log phase cultures of TIGR4, Δ*potABCD* and the complement pABG5-*potABCD* strain grown in CDM (OD600 0.4–0.5) were centrifuged at 10,000× *g* for 2 min and cells were suspended in PBS. The cells (10^8^ CFU/mL) (1 mL PBS) were then supplemented with final concentrations of hydrogen peroxide 2.5 mM and incubated at 37 °C with 5% CO_2_ for 15 min. CDM is devoid of polyamines but has the amino acid precursors for polyamine synthesis. To determine the effects of polyamines on pneumococcal H_2_O_2_ stress, Δ*potABCD* challenged with 2.5 mM H_2_O_2_, was supplemented with either cadaverine or agmatine (½MIC, ¼MIC, ⅛MIC) and incubated for 15 min. Control reactions had untreated bacteria, and CFUs were determined by serial dilution in PBS and plating on BAP. Results from three independent experiments were expressed as survival percentage of treated bacteria relative to the untreated bacteria.

### 4.10. S-Nitrosoglutathione (GSNO) Susceptibility

Mid-log phase cultures of TIGR4, Δ*potABCD*, and the complement pABG5-*potABCD* strain grown in CDM were centrifuged at 10,000× *g* for 2 min and cells were suspended in PBS. The cells (10^7^ CFU/mL) in 100 µL were supplemented with a final concentration of 2.5 mM GSNO (Sigma-Aldrich, Israel), a nitric oxide producer, and incubated at 37 °C in 5% CO_2_ for 60 min. In addition, Δ*potABCD* challenged with 2.5 mM GSNO was supplemented with either cadaverine or agmatine (½MIC, ¼MIC, ⅛MIC). Control reactions had untreated bacteria, and CFUs were determined by serial dilution in PBS and plating on BAP every after 15 min. Results from three independent experiments were expressed as percent survival of treated bacteria relative to the untreated bacteria.

### 4.11. Statistical Analysis

Significant differences between the susceptibility of TIGR4, Δ*potABCD* and the complement pABG5-*potABCD* strain to the different stressors, changes in pH_i,_ production of endogenous H_2_O_2_, levels of NADPH and GSH/GSSG ratio, as well as changes in gene expression measured by qRT-PCR were determined by Student’s *t*-test at *p* ≤ 0.05.

## Figures and Tables

**Table 1:** The *potABCD* operon is essential for pneumococcal stress responses.

Locus Tag	Gene	Fold Change Δ*potABCD*/TIGR4	FDR *p* Value	Description
SP_1884	SP_1884	−3.6	<0.0001	PTS trehalose transporter subunit IIBC
SP_1883	SP_1883	−3.2	<0.0001	Trehalose 6-phosphate hydrolase
SP_1907	*groES*	−3.9	<0.0001	Co-chaperone
SP_0519	*dnaJ*	−3.8	<0.0001	Molecular chaperone
SP_0517	*dnaK*	−4.2	<0.0001	Molecular chaperone
SP_1906	*groEL*	−3.3	<0.0001	Molecular chaperone
SP_1870	SP_1870	2.7	<0.0001	Iron-compound ABC transporter
SP_1871	SP_1871	2.9	<0.0001	Iron ABC transporter ATP-binding protein
SP_1241	SP_1241	3.1	<0.0001	Glutamine transport system substrate-binding
SP_1242	SP_1242	3.2	<0.0001	Glutamine transport system ATP-binding protein
SP_2087	*pstB*	41.8	<0.0001	Phosphate ABC transporter ATP-binding protein
SP_2085	*pstC*	37.2	<0.0001	Phosphate ABC transporter permease subunit
SP_2084	*pstS*	27.2	<0.0001	Phosphate ABC transporter substrate-binding
SP_2086	*pstA*	40.6	<0.0001	Phosphate ABC transporter permease protein
SP_1650	*psaA*	3.2	<0.0001	Manganese ABC transporter substrate-binding
SP_1648	*psaB*	3.4	<0.0001	Metal ABC transporter ATP-binding protein
SP_1649	*psaC*	3.5	<0.0001	Metal ABC transporter permease
SP_0502	*glnA*	4.2	<0.0001	Glutamine synthetase
SP_2148	*arcA*	4.6	<0.0001	Arginine deiminase
SP_2151	*arcC*	3.7	<0.0001	Carbamate kinase
SP_0798	*ciaR*	3.0	<0.0001	DNA-binding response regulator
SP_0799	*ciaH*	3.0	<0.0001	Two-component sensor histidine kinase
SP_0501	*merR*	4.0	<0.0001	MerR family transcriptional regulator
SP_0515	*hrcA*	−4.3	<0.0001	Transcriptional regulator
SP_2088	*phoU*	47.2	<0.0001	Phosphate uptake regulator

FDR (False discovery rate).

**Table 2:** The *potABCD* operon promotes galactose utilization and the pentose phosphate pathway.

Locus Tag	Gene	Fold Change Δ*potABCD*/TIGR4	FDR *p* Value	Description
SP_2127	*tktN*	222.7	<0.0001	Transketolase
SP_2128	*tktC*	225.5	<0.0001	Transketolase
SP_2130	*SP_2130*	211.5	<0.0001	Ascorbate PTS system EIIB
SP_2129	*SP_2129*	214.7	<0.0001	PTS ascorbate transporter subunit IIC
SP_1192	*lacB*	2.8	<0.0001	Galactose 6-phosphate isomerase subunit
SP_1193	*lacA*	2.8	<0.0001	Galactose 6-phosphate isomerase subunit
SP_1190	*lacD*	2.8	<0.0001	Tagatose 1,6-diphosphate aldolase
SP_1191	*lacC*	2.8	<0.0001	Tagatose 6-phosphate kinase
SP_1186	*lacF-2*	2.0	<0.0001	Lactose PTS system EIIA component
SP_2165	*fucU*	4.5	<0.0001	Fucose isomerase
SP_2166	*fucA*	3.8	<0.0001	L-fuculose phosphate aldolase
SP_1853	*galK*	6.1	<0.0001	Galactokinase
SP_0066	*galM*	2.7	<0.0001	Galactose mutarotase
SP_0064	SP_0064	3.4	<0.0001	PTS mannose PTS system EIIA
SP_0645	SP_0645	8.8	<0.0001	PTS galactose PTS system EIIA
SP_0646	SP_0646	9.0	<0.0001	PTS galactose PTS system EIIB
SP_2164	SP_2164	3.7	<0.0001	PTS mannose transporter subunit IIA
SP_2161	SP_2161	3.3	<0.0001	PTS mannose transporter subunit IID
SP_2162	SP_2162	3.7	<0.0001	PTS mannose PTS system EIIC

FDR (False discovery rate).

**Table 3:** The *potABCD* operon modulates central metabolism and production of precursors for the pneumococcal capsule.

Locus Tag	Gene	Fold Change Δ*potABCD*/TIGR4	FDR *p* Value	Description
SP_2131	*bglG*	247.3	<0.0001	Transcriptional regulator
SP_0100	*padR*	6.8	<0.0001	Transcriptional regulator
SP_1854	*galR*	3.9	<0.0001	LacI family transcriptional regulator
SP_2109	*malC*	−2.5	<0.0001	Maltodextrin ABC transporter permease
SP_2110	*malD*	−2.4	<0.0001	Maltodextrin ABC transporter permease
SP_2108	*malX*	−2.4	<0.0001	Maltose/maltodextrin-binding protein
SP_1894	*gtfA*	2.3	<0.0001	Sucrose phosphorylase
SP_1722	SP_1722	−51.0	<0.0001	PTS sucrose system EIIBCA or EIIBC
SP_0648	*bgaA*	7.0	<0.0001	Beta galactosidase
SP_1898	*aga*	2.2	<0.0001	Alpha galactosidase
SP_1721	*scrK*	−10.0	<0.0001	Fructokinase
SP_1725	*scrR*	−9.2	<0.0001	LacI family transcriptional regulator,
SP_1278	*pyrR*	−2.7	<0.0001	Bifunctional pyrimidine operon transcriptional regulator
SP_1724	*scrB*	−10.5	<0.0001	Sucrose 6-phosphate hydrolase
SP_1415	*nagB*	3.2	<0.0001	Glucosamine 6-phosphate deaminase
SP_2056	*nagA*	2.3	<0.0001	N-acetylglucosamine 6-phosphate deacetylase
SP_0321	SP_0321	−2.3	<0.0001	PTS N-acetylgalactosamine transporter subunit IIA
SP_0323	SP_0323	−2.6	<0.0001	PTS N-acetylgalactosamine PTS system EIIB
SP_1277	*pyrB*	−2.4	<0.0001	Aspartate carbamoyltransferase
SP_1014	*dapA*	−4.3	<0.0001	4-hydroxy-tetrahydrodipicolinate synthase
SP_1013	*asd*	−5.5	<0.0001	Aspartate-semialdehyde dehydrogenase

FDR (False discovery rate).

**Table 4:** Significant changes in metabolites in response to *potABCD* operon deletion.

Locus Tag	Gene	Fold Change Δ*potABCD*/TIGR4	*p* Value	Description
SP_0916	*speA*	16.9	<0.0001	Arginine decarboxylase
SP_0918	*speE*	14.4	<0.0001	Spermidine synthase
SP_2127	SP_2127	20.3	<0.0001	Transketolase C-terminal subunit
SP_2128	SP_2128	27.7	<0.0001	Transketolase N-terminal subunit
SP_2131	*BglG*	5.5	<0.0001	Transcriptional regulator
SP_2136	*pcpA*	7.2	<0.0001	Choline-binding protein
SP_1650	*psaA*	5.40	<0.0001	Manganese ABC transporter

**Table 5:** Significant changes in Δ*potABCD* gene expression compared with TIGR4 measured by qRT-PCR.

Locus Tag	Gene	Fold Change Δ*potABCD*/TIGR4	*p* Value	Description
SP_0916	*speA*	16.9	<0.0001	Arginine decarboxylase
SP_0918	*speE*	14.4	<0.0001	Spermidine synthase
SP_2127	SP_2127	20.3	<0.0001	Transketolase, C-terminal subunit
SP_2128	SP_2128	27.7	<0.0001	Transketolase, N-terminal subunit
SP_2131	*BglG*	5.5	<0.0001	Transcriptional regulator
SP_2136	*pcpA*	7.2	<0.0001	Choline-binding protein
SP_1650	*psaA*	5.40	<0.0001	Manganese ABC transporter

## Data Availability

RNA-Seq raw data and metadata are available at NCBI GEO with the accession number PRJNA722029. https://www.ncbi.nlm.nih.gov/bioproject/?term=Pneumococcal.

## Supplementary Materials

The following are available online at https://www.mdpi.com/article/10.3390/pathogens10101322/s1. Table S1: Sequences of primers used in this study. Table S2: Significant changes in gene expression between Δ*potABCD* and TIGR4 that are not included in the tables in the text.
