# Supplementary material for: The Effect of Impaired Polyamine Transport on Pneumococcal Transcriptome
Source: Pathogens. 2021 Oct 14;10(10):1322. doi: 10.3390/pathogens10101322 (PMC8540371; doi:10.3390/pathogens10101322)
Supplement: Supplementary file 1 [file pathogens-10-01322-s001.zip › Table S1.pdf]

Table S1. Sequences of primers used in this study

Primers	Sequence (5'→3')
speA	Rev- AAACCGAAACACCGTCTATTAACTC
speE	For-TGCGGATGATTTCGTCTACAATG
	Rev-GCTTAGTCCTGGTCGCAATC
SP_2127	For- TGCAGCAGGCTTGTCTATTC
	Rev- GGCATCCAATTGTGCGTATC
SP_2128	For- TATGGTCAGAGAATAAGAAAGAGTCC
	Rev- GTTGGATAACTGTTGATGAGAAGC
bglG	For- TGAGAACTACCCTGAGGCAATG
	Rev- ATTCCTATTTCATTCACACCTTCATC
pcpA	For- TTCTTAATATGAATTTCCTTTAATG
	Rev- GGTAATAATATCAACTCATTGC
