# Supplementary material for: The Effect of Impaired Polyamine Transport on Pneumococcal Transcriptome
Source: Pathogens. 2021 Oct 14;10(10):1322. doi: 10.3390/pathogens10101322 (PMC8540371; doi:10.3390/pathogens10101322)
Supplement: Supplementary file 1 [file pathogens-10-01322-s001.zip › Table S2.pdf]

Table S2. Significant changes in gene expression between *ΔpotABCD* and TIGR4 that are not included in the tables in the text.

Description	Locus tag	Fold change	FDR p-value
ABC transporter permease	SP_1388	-484.5	>0.0001
ABC transporter permease	SP_1387	-378	>0.0001
Spermidine/putrescine ABC transporter substrate-binding protein	SP_1386	-349.2	>0.0001
Spermidine/putrescine import ATP-binding protein PotA	SP_1389	-337.4	>0.0001
PTS beta-glucoside transporter subunit IIBCA	SP_1722	-51	>0.0001
Guanosine monophosphate reductase	SP_1249	-11.1	>0.0001
Sucrose-6-phosphate hydrolase	SP_1724	-10.5	>0.0001
Fructokinase	SP_1721	-10	>0.0001
tRNA-Arg	SP_2249	-9.8	>0.0001
LacI family transcriptional regulator	SP_1725	-9.2	>0.0001
Aspartate-semialdehyde dehydrogenase	SP_1013	-5.5	>0.0001
Pyridoxal 5'-phosphate synthase glutaminase subunit PdxT	SP_1467	-5.1	>0.0001
Pyridoxal 5'-phosphate synthase lyase subunit PdxS	SP_1468	-4.7	>0.0001
Trehalose operon repressor	SP_1885	-4.7	>0.0001
4-hydroxy-tetrahydrodipicolinate synthase	SP_1014	-4.3	>0.0001
Nucleotide exchange factor GrpE	SP_0516	-4.1	>0.0001
Co-chaperone GroES	SP_1907	-3.9	>0.0001
tRNA-Ser	SP_2253	-3.9	>0.0001
tRNA-Ser	SP_2253	-3.9	>0.0001
Transcriptional regulator	SP_1131	-3.6	>0.0001
PTS trehalose transporter subunit IIBC	SP_1884	-3.6	>0.0001
Alpha-amylase	SP_1382	-3.5	>0.0001
tRNA-Gly	SP_2264	-3.5	>0.0001
tRNA-Gly	SP_2264	-3.5	>0.0001
tRNA-Arg	SP_2262	-3.4	>0.0001
tRNA-Arg	SP_2262	-3.4	>0.0001
tRNA-Arg	SP_2274	-3.4	>0.0001
tRNA-Arg	SP_2274	-3.4	>0.001
Alpha%2Calpha-phosphotrehalase	SP_1883	-3.2	>0.001
ECF transporter S component	SP_1597	-3	>0.001
tRNA-Met	SP_2260	-3	>0.001
Maltose PTS system EIICB or EIICBA component	SP_0758	-2.9	>0.001
Hypothetical protein	SP_1006	-2.9	>0.001
tRNA-Leu	SP_2263	-2.9	>0.001
tRNA-Leu	SP_2263	-2.9	>0.001
tRNA-Leu	SP_2275	-2.9	>0.001

tRNA-Leu	SP_2275	-2.9	>0.001
tRNA-Gly	SP_2276	-2.9	>0.001
tRNA-Gly	SP_2276	-2.9	>0.001
Hypothetical protein	SP_1007	-2.8	>0.001
tRNA-Arg	SP_2250	-2.8	>0.001
tRNA-Pro	SP_2273	-2.8	>0.001
tRNA-Pro	SP_2273	-2.8	>0.001
Hypothetical protein	SP_1004	-2.7	>0.001
Bifunctional pyrimidine operon transcriptional regulator/uracil phosphoribosyltransferase	SP_1278	-2.7	>0.001
tRNA-Lys	SP_2267	-2.7	>0.001
tRNA-Leu	SP_2284	-2.7	>0.001
tRNA-Gly	SP_2293	-2.7	>0.001
Type I addiction module toxin%2C Fst family	SP_0258	-2.6	>0.001
PTS system mannose/fructose/N-acetylgalactosamine-Transporter subunit IIB	SP_0323	-2.6	>0.001
Pyridoxine kinase	SP_1598	-2.6	>0.001
tRNA-Gln	SP_2246	-2.6	>0.001
tRNA-Pro	SP_2261	-2.6	>0.001
tRNA-Thr	SP_2265	-2.6	>0.001
tRNA-Thr	SP_2277	-2.6	>0.001
tRNA pseudouridine(38-40) synthase TruA	SP_1599	-2.5	>0.001
Maltodextrin ABC transporter permease	SP_2109	-2.5	>0.001
tRNA-Gly	SP_2255	-2.5	>0.001
tRNA-Lys	SP_2279	-2.5	>0.001
tRNA-Trp	SP_2287	-2.5	>0.001
tRNA-Glu	SP_2296	-2.5	>0.001
Aspartate carbamoyltransferase	SP_1277	-2.4	>0.001
Maltodextrin phosphorylase	SP_2106	-2.4	>0.001
Maltose/maltodextrin-binding protein	SP_2108	-2.4	>0.001
Maltodextrin ABC transporter permease	SP_2110	-2.4	>0.001
RNA-His	SP_2286	-2.4	>0.001
PTS N-acetylgalactosamine transporter subunit IIA	SP_0321	-2.3	>0.001
tRNA-Leu	SP_2266	-2.3	>0.001
tRNA-Leu	SP_2278	-2.3	>0.001
hydrolase	SP_0760	-2.2	>0.001
tRNA-Ile	SP_2292	-2.2	>0.001
Carbamoyl-phosphate synthase small chain	SP_1276	-2.1	>0.001
tRNA-Ile	SP_2254	-2.1	>0.001
tRNA-Tyr	SP_2288	-2.1	>0.001
N-acetylgalactosamine PTS system EIID component	SP_0325	-2	>0.001

Phenylalanine--tRNA ligase subunit alpha	SP_0579	-2	>0.001
N-acetyltransferase	SP_0580	-2	>0.001
Hypothetical protein	SP_1039	-2	>0.001
FtsW/RodA/SpoVE family cell cycle protein	SP_1067	-2	>0.001
tRNA-Phe	SP_2256	-2	>0.001
tRNA-Met	SP_2259	-2	>0.001
tRNA-Phe	SP_2289	-2	>0.001
Adenylosuccinate synthetase	SP_0019	-1.9	>0.001
Preprotein translocase subunit YajC	SP_0326	-1.9	0.005
Orotidine-5'-phosphate decarboxylase	SP_0701	-1.9	>0.001
Pyruvate dehydrogenase E2 component (dihydrolipoamide acetyltransferase	SP_1162	-1.9	0.003
Thiamine pyrophosphate-dependent dehydrogenase E1 component subunit alpha	SP_1164	-1.9	0.002
Carbamoyl-phosphate synthase large chain	SP_1275	-1.9	>0.001
Collagenase-like protease	SP_1429	-1.9	>0.001
Hypothetical protein	SP_1494	-1.9	0.017
Type 2 lantibiotic	SP_1948	-1.9	>0.001
Type 2 lantibiotic	SP_1949	-1.9	>0.001
tRNA-Gln	SP_2285	-1.9	>0.001
Transporter ComB	SP_0043	-1.8	>0.001
MetQ/NlpA family ABC transporter substrate-binding protein	SP_0149	-1.8	>0.001
NCS2 family permease	SP_0287	-1.8	>0.001
CPBP family intramembrane metalloprotease	SP_0288	-1.8	>0.001
Unsaturated chondroitin disaccharide hydrolase	SP_0322	-1.8	0.002
Glucan 1%2C6-alpha-glucosidase	SP_0342	-1.8	>0.001
Hypothetical protein	SP_0451	-1.8	>0.001
Helicase	SP_0564	-1.8	>0.001
Orotate phosphoribosyltransferase	SP_0702	-1.8	>0.001
Saccharopine dehydrogenase	SP_0919	-1.8	>0.001
Lactoylglutathione lyase	SP_0962	-1.8	>0.001
Peptidase T	SP_1008	-1.8	>0.001
Phosphoenolpyruvate carboxylase	SP_1068	-1.8	>0.001
HU family DNA-binding protein	SP_1113	-1.8	0.026
Alpha-ketoacid dehydrogenase subunit beta	SP_1163	-1.8	0.004
chorismate mutase	SP_1296	-1.8	0.002
30S ribosomal protein S21	SP_1414	-1.8	0.017
Hypothetical protein	SP_1427	-1.8	>0.001
GMP synthase (glutamine-hydrolyzing)	SP_1445	-1.8	>0.001
Dead/Deah box family ATP-dependent RNA helicase	SP_1586	-1.8	0.002
tRNA guanosine (34) transglycosylase Tgt	SP_2058	-1.8	>0.001
4-alpha-glucanotransferase	SP_2107	-1.8	0.003

Bacteriocin cleavage/export ABC transporter	SP_0042	-1.7	>0.001
Rhodanese domain-containing protein	SP_0095	-1.7	>0.001
N-acetylgalactosamine PTS system EIIC component	SP_0324	-1.7	0.022
Phenylalanine--tRNA ligase subunit beta	SP_0581	-1.7	>0.001
Polyamine aminopropyltransferase	SP_0918	-1.7	>0.001
N-carbamoylputrescine amidase	SP_0922	-1.7	>0.001
ABC transporter ATP-binding protein	SP_1035	-1.7	0.010
Lipoate--protein ligase A	SP_1160	-1.7	>0.001
Dihydrolipoyl dehydrogenase	SP_1161	-1.7	>0.001
Noncanonical pyrimidine nucleotidase%2C YjjG family	SP_1171	-1.7	>0.001
Fluoride exporter	SP_1295	-1.7	>0.001
Hypothetical protein	SP_1493	-1.7	>0.001
Amino acid ABC transporter permease	SP_1502	-1.7	>0.001
XRE family transcriptional regulator	SP_1946	-1.7	>0.001
Ribonuclease P protein component	SP_2042	-1.7	>0.001
Hypothetical protein	SP_2199	-1.7	0.002
Hypothetical protein	SP_0122	-1.6	>0.001
PTS mannose transporter subunit IIAB	SP_0284	-1.6	0.019
30S ribosomal protein S9	SP_0295	-1.6	>0.001
Serine--tRNA ligase	SP_0411	-1.6	>0.001
Amino acid ABC transporter ATP-binding protein	SP_0452	-1.6	>0.001
Glutamine ABC transporter substrate-binding protein	SP_0453	-1.6	0.025
tRNA (cytidine(34)-2'-O)-methyltransferase	SP_0486	-1.6	>0.001
CTP synthetase	SP_0494	-1.6	>0.001
Aminoglycoside phosphotransferase	SP_0549	-1.6	>0.001
Ribosome maturation factor RimP	SP_0552	-1.6	>0.001
Hypothetical protein	SP_0555	-1.6	>0.001
Ribosome-binding factor A	SP_0557	-1.6	0.021
5-methyltetrahydropteroyltriglutamate--homocysteine methyltransferase	SP_0585	-1.6	>0.001
Glucokinase	SP_0668	-1.6	>0.001
30S ribosomal protein S20	SP_0838	-1.6	0.041
Aminotransferase class V-fold PLP-dependent enzyme	SP_0916	-1.6	>0.001
Carboxynorspermidine decarboxylase	SP_0920	-1.6	>0.001
Agmatine deiminase	SP_0921	-1.6	>0.001
Peptide ABC transporter substrate-binding protein	SP_1069	-1.6	>0.001
Type II toxin-antitoxin system RelE/ParE family toxin	SP_1223	-1.6	0.002
Uracil transporter	SP_1286	-1.6	>0.001
50S ribosomal protein L10	SP_1355	-1.6	>0.001
Transcription elongation factor GreA	SP_1517	-1.6	>0.001
Ferredoxin--NADP(+) reductase	SP_1563	-1.6	>0.001
MFS transporter	SP_1587	-1.6	>0.001

UDP-glucose 4-epimerase GalE	SP_1607	-1.6	>0.001
2-Cys peroxiredoxin	SP_1651	-1.6	0.024
Beta-fructofuranosidase	SP_1795	-1.6	0.017
Hypothetical protein	SP_1945	-1.6	0.004
Glycoside hydrolase family 1 protein	SP_2021	-1.6	0.013
PTS sugar transporter subunit IIB	SP_2023	-1.6	0.003
PTS lactose/cellobiose transporter subunit IIA	SP_2024	-1.6	0.005
Glutamate--tRNA ligase	SP_2069	-1.6	>0.001
Maltodextrose utilization protein MalA	SP_2111	-1.6	>0.001
Hypothetical protein	SP_2115	-1.6	0.003
Transcriptional regulator	SP_2172	-1.6	>0.001
Elongation factor Ts	SP_2214	-1.6	0.001
tRNA-Met	SP_2257	-1.6	0.002
Bifunctional 3%2C4-dihydroxy-2-butanone-4-phosphate synthase/GTP cyclohydrolase II	SP_0176	-1.5	0.002
Hypothetical protein	SP_0198	-1.5	0.007
Adenylate kinase	SP_0231	-1.5	>0.001
50S ribosomal protein L13	SP_0294	-1.5	0.008
HIT family protein	SP_0521	-1.5	>0.001
tRNA (guanosine(46)-N7)-methyltransferase TrmB	SP_0550	-1.5	>0.001
Transcription termination/antitermination protein NusA	SP_0553	-1.5	>0.001
DUF448 domain-containing protein	SP_0554	-1.5	>0.001
Translation initiation factor IF-2	SP_0556	-1.5	0.005
DUF3042 domain-containing protein	SP_0670	-1.5	>0.001
Translational GTPase TypA	SP_0681	-1.5	0.003
30S ribosomal protein S16	SP_0775	-1.5	0.042
KH domain-containing protein	SP_0776	-1.5	0.013
DUF2829 domain-containing protein	SP_0792	-1.5	0.004
30S ribosomal protein S1	SP_0862	-1.5	>0.001
AbrB family transcriptional regulator	SP_0888	-1.5	0.009
Integrase	SP_0890	-1.5	0.021
Iron ABC transporter permease	SP_1033	-1.5	>0.001
ABC transporter permease	SP_1070	-1.5	>0.001
Uridine kinase	SP_1208	-1.5	>0.001
DUF454 domain-containing protein	SP_1261	-1.5	0.003
Bifunctional oligoribonuclease/PAP phosphatase NrnA	SP_1298	-1.5	>0.001
50S ribosomal protein L7/L12	SP_1354	-1.5	0.005
Chlorohydrolase	SP_1356	-1.5	>0.001
Regulatory protein Spx	SP_1405	-1.5	0.002
Glutamine ABC transporter substrate-binding protein	SP_1500	-1.5	>0.001
Amino acid ABC transporter ATP-binding protein	SP_1501	-1.5	>0.001
30S ribosomal protein S15	SP_1626	-1.5	0.012

Oxidoreductase	SP_1686	-1.5	0.019
Aquaporin	SP_1778	-1.5	0.008
Hypothetical protein	SP_1794	-1.5	0.049
Hypothetical protein	SP_1882	-1.5	0.005
PTS sugar transporter subunit IIC	SP_2022	-1.5	0.015
Transketolase	SP_2030	-1.5	0.002
Cytidine deaminase	SP_2068	-1.5	>0.001
Transcriptional regulator	SP_2112	-1.5	>0.001
Transcriptional regulator	SP_2119	-1.5	0.008
Zinc transport system ATP-binding protein AdcC	SP_2171	-1.5	>0.001
ABC transporter ATP-binding protein	SP_2196	-1.5	>0.001
ABC transporter substrate-binding protein	SP_2197	-1.5	>0.001
tRNA-Ser	SP_2258	-1.5	0.002
tRNA-Met	SP_2290	-1.5	0.006
tRNA-Ser	SP_2291	-1.5	0.004
DNA polymerase III subunit beta	SP_0002	-1.4	>0.001
Peptidyl-tRNA hydrolase	SP_0005	-1.4	0.005
Carbonic anhydrase	SP_0024	-1.4	>0.001
PrsW family intramembrane metalloprotease	SP_0026	-1.4	0.004
Amino acid ABC transporter substrate-binding protein	SP_0148	-1.4	0.001
50S ribosomal protein L36	SP_0233	-1.4	0.003
30S ribosomal protein S13	SP_0234	-1.4	0.030
30S ribosomal protein S11	SP_0235	-1.4	0.022
PFL family protein	SP_0239	-1.4	0.002
LLM class flavin-dependent oxidoreductase	SP_0267	-1.4	0.003
DUF956 domain-containing protein	SP_0412	-1.4	0.003
Elongation factor P	SP_0435	-1.4	>0.001
DNA-directed RNA polymerase subunit delta	SP_0481	-1.4	0.003
DUF3816 family protein	SP_0482	-1.4	0.002
Aromatic acid exporter family protein	SP_0500	-1.4	0.003
Hypothetical protein	SP_0520	-1.4	0.010
Oxidoreductase	SP_0606	-1.4	0.001
DUF910 domain-containing protein	SP_0677	-1.4	0.024
DUF1027 domain-containing protein	SP_0767	-1.4	0.002
Hypothetical protein	SP_0782	-1.4	0.004
3-oxoacyl-ACP reductase	SP_0793	-1.4	0.011
U32 family peptidase	SP_0801	-1.4	0.001
BMP family ABC transporter substrate-binding protein	SP_0845	-1.4	0.031
DNA topoisomerase IV subunit B	SP_0852	-1.4	>0.001
N-acetyltransferase	SP_0953	-1.4	0.007
50S ribosomal protein L35	SP_0960	-1.4	>0.001
50S ribosomal protein L20	SP_0961	-1.4	0.008

Dihydroorotate dehydrogenase electron transfer subunit	SP_0963	-1.4	0.001
Preprotein translocase subunit SecG	SP_0974	-1.4	0.037
SsrA-binding protein	SP_0976	-1.4	>0.001
Tellurite methyltransferase	SP_0977	-1.4	>0.001
Thymidine kinase	SP_1018	-1.4	>0.001
Iron ABC transporter permease	SP_1034	-1.4	>0.001
Phosphonate ABC transporter ATP-binding protein	SP_1071	-1.4	0.013
Hypothetical protein	SP_1080	-1.4	0.008
CYTH domain-containing protein	SP_1096	-1.4	0.014
ABC transporter ATP-binding protein	SP_1114	-1.4	0.004
Site-specific integrase	SP_1129	-1.4	>0.001
Hypothetical protein	SP_1140	-1.4	0.026
L-lactate dehydrogenase	SP_1220	-1.4	0.034
Phosphopantothenate--cysteine ligase	SP_1230	-1.4	0.032
Fluoride exporter	SP_1294	-1.4	0.006
Class I SAM-dependent rRNA methyltransferase	SP_1378	-1.4	0.005
PLP-dependent aminotransferase family protein	SP_1393	-1.4	0.003
GntR family transcriptional regulator	SP_1446	-1.4	0.009
NADH oxidase	SP_1469	-1.4	0.038
DUF896 domain-containing protein	SP_1473	-1.4	0.005
Glycine--tRNA ligase subunit beta	SP_1474	-1.4	0.005
Glycine--tRNA ligase subunit alpha	SP_1475	-1.4	>0.001
ATP synthase subunit delta	SP_1511	-1.4	0.005
Endolytic transglycosylase MltG	SP_1518	-1.4	0.016
Aminopeptidase P family protein	SP_1591	-1.4	0.002
Hypothetical protein	SP_1602	-1.4	0.004
Glycosyltransferase	SP_1606	-1.4	>0.001
1-acyl-sn-glycerol-3-phosphate acyltransferase	SP_1624	-1.4	0.007
2%2C3-bisphosphoglycerate-dependent phosphoglycerate mutase	SP_1655	-1.4	0.042
Isoleucine--tRNA ligase	SP_1659	-1.4	0.015
Thioredoxin	SP_1776	-1.4	0.010
DUF421 domain-containing protein	SP_1841	-1.4	0.030
DUF3290 domain-containing protein	SP_1844	-1.4	0.009
Asparagine synthetase A	SP_1970	-1.4	0.007
C4-dicarboxylate ABC transporter	SP_2017	-1.4	0.005
Glycerol-3-phosphate dehydrogenase (NAD(P)(+))	SP_2091	-1.4	>0.001
Tyrosine--tRNA ligase	SP_2100	-1.4	0.011
30S ribosomal protein S2	SP_2215	-1.4	0.015
Chromosomal replication initiator protein DnaA	SP_0001	-1.3	0.003
Redox-regulated ATPase YchF	SP_0004	-1.3	0.030
Histidine phosphatase family protein	SP_0022	-1.3	0.029
GntR family transcriptional regulator	SP_0058	-1.3	0.007

Hypothetical protein	SP_0142	-1.3	0.041
Peptidase M20	SP_0150	-1.3	0.024
Flavoprotein NrdI	SP_0158	-1.3	>0.001
Bifunctional diaminohydroxyphosphoribosylaminopyrimidine Deaminase/5-amino-6-(5-phosphoribosylamino) uracil reductase RibD	SP_0178	-1.3	0.020
LD-carboxypeptidase	SP_0182	-1.3	>0.001
Translation initiation factor IF-1	SP_0232	-1.3	0.050
DNA-directed RNA polymerase subunit alpha	SP_0236	-1.3	0.034
Carboxymuconolactone decarboxylase family protein	SP_0409	-1.3	0.034
N utilization substance protein B	SP_0433	-1.3	0.007
Asp23/Gls24 family envelope stress response protein	SP_0434	-1.3	0.009
Asp-Trna (Asn)/Glu-tRNA (Gln) amidotransferase GatCAB subunit C	SP_0438	-1.3	0.016
Thymidylate synthase	SP_0669	-1.3	>0.001
DUF3165 domain-containing protein	SP_0682	-1.3	0.025
Transcriptional regulator	SP_0716	-1.3	0.011
Hydroxyethylthiazole kinase	SP_0717	-1.3	0.039
Branched-chain amino acid ABC transporter substrate-binding protein	SP_0749	-1.3	0.007
Branched-chain amino acid ABC transporter permease	SP_0750	-1.3	0.010
ABC transporter ATP-binding protein	SP_0753	-1.3	0.027
Peptidylprolyl isomerase	SP_0771	-1.3	0.012
Hypothetical protein	SP_0781	-1.3	0.027
Fe-S cluster assembly ATPase SufC	SP_0867	-1.3	0.028
Alpha/beta hydrolase	SP_0882	-1.3	0.041
LysR family transcriptional regulator	SP_0927	-1.3	0.043
FADH (2)-oxidizing methylenetetrahydrofolate--tRNA-(uracil(54)-C (5))-methyltransferase TrmFO	SP_0943	-1.3	0.007
Ribonuclease R	SP_0975	-1.3	0.054
Bifunctional N-acetylglucosamine-1-phosphate Uridyltransferase/glucosamine-1-phosphate acetyltransferase	SP_0988	-1.3	0.023
Hypothetical protein	SP_1003	-1.3	0.038
Tautomerase	SP_1017	-1.3	0.036
Serine recombinase	SP_1040	-1.3	0.027
MFS transporter	SP_1116	-1.3	0.004
Uracil-DNA glycosylase	SP_1169	-1.3	0.023
Type II restriction endonuclease	SP_1221	-1.3	0.016
Type II restriction endonuclease	SP_1222	-1.3	>0.001
3-isopropylmalate dehydrogenase	SP_1250	-1.3	0.048
Endonuclease	SP_1251	-1.3	0.027
Endonuclease III	SP_1279	-1.3	>0.001
Zinc metalloprotease HtpX	SP_1283	-1.3	0.018
LemA family protein	SP_1284	-1.3	>0.001
Flavodoxin	SP_1297	-1.3	0.034
UDP-N-acetylenolpyruvoylglucosamine reductase	SP_1384	-1.3	0.005

ATP synthase epsilon chain	SP_1507	-1.3	0.030
ATP synthase subunit alpha	SP_1510	-1.3	0.039
ATP synthase subunit C	SP_1514	-1.3	0.049
N-acetyltransferase	SP_1516	-1.3	0.011
Manganese-dependent inorganic pyrophosphatase	SP_1534	-1.3	0.026
Hypothetical protein	SP_1537	-1.3	0.019
NAD(P)H-dependent oxidoreductase	SP_1546	-1.3	0.002
Cation transporter	SP_1552	-1.3	>0.001
1%2C4-beta-N-acetylmuramidase	SP_1573	-1.3	0.052
Triose-phosphate isomerase	SP_1574	-1.3	0.032
MFS transporter	SP_1600	-1.3	0.039
Hypothetical protein	SP_1604	-1.3	0.009
tRNA (adenine-N(1))-methyltransferase	SP_1610	-1.3	0.032
ISL3 family transposase	SP_1692	-1.3	0.037
Aromatic acid exporter family protein	SP_1754	-1.3	0.012
Replication-associated recombination protein A	SP_1790	-1.3	0.015
UTP--glucose-1-phosphate uridylyltransferase	SP_1867	-1.3	0.013
membrane protein insertion efficiency factor YidD	SP_1873	-1.3	>0.001
Membrane protein	SP_2027	-1.3	0.024
Low molecular weight phosphotyrosine protein phosphatase	SP_2028	-1.3	0.004
Class I SAM-dependent methyltransferase	SP_2045	-1.3	0.005
Histidine--tRNA ligase	SP_2121	-1.3	0.021
SPFH domain-containing protein	SP_2156	-1.3	0.019
Zinc-binding lipoprotein AdcA	SP_2169	-1.3	>0.001
ABC transporter permease	SP_2198	-1.3	0.012
Ribose-phosphate pyrophosphokinase	SP_0031	1.3	0.038
L-serine ammonia-lyase%2C iron-sulfur-dependent%2C subunit alpha	SP_0105	1.3	0.009
Lactococcin 972 family bacteriocin	SP_0109	1.3	0.038
Amino acid ABC transporter permease	SP_0110	1.3	0.004
Glycoside hydrolase	SP_0135	1.3	0.037
DUF624 domain-containing protein	SP_0154	1.3	0.027
Hypothetical protein	SP_0157	1.3	0.024
Excinuclease ABC subunit A	SP_0186	1.3	0.002
Hypothetical protein	SP_0352	1.3	0.017
Restriction endonuclease subunit S	SP_0505	1.3	0.044
UvrABC system protein C	SP_0618	1.3	>0.001
Metallophosphoesterase	SP_0619	1.3	>0.001
Sensor histidine kinase	SP_0662	1.3	0.008
Thiamine/molybdopterin biosynthesis protein	SP_0695	1.3	0.034
ATP-dependent Clp protease ATP-binding subunit	SP_0820	1.3	0.042
DNA polymerase III subunit alpha	SP_0895	1.3	0.020
Endo-beta-N-acetylglucosaminidase	SP_0965	1.3	>0.001

RNA methyltransferase	SP_1029	1.3	0.009
XRE family transcriptional regulator	SP_1144	1.3	0.032
ATP-dependent helicase/nuclease subunit A	SP_1152	1.3	0.018
Hypothetical protein	SP_1153	1.3	>0.001
Glucose-6-phosphate 1-dehydrogenase	SP_1243	1.3	0.042
Signal recognition particle-docking protein FtsY	SP_1244	1.3	0.027
Cof-type HAD-IIB family hydrolase	SP_1246	1.3	0.003
Choline kinase	SP_1269	1.3	0.024
DNA-binding protein	SP_1292	1.3	0.035
MBL fold metallo-hydrolase	SP_1646	1.3	0.002
MurR/RpiR family transcriptional regulator	SP_1674	1.3	>0.001
Hypothetical protein	SP_1728	1.3	0.014
Primosomal protein N'	SP_1736	1.3	0.019
ImmA/IrrE family metallo-endopeptidase	SP_1809	1.3	0.010
Polyketide cyclase	SP_1862	1.3	0.008
MarR family transcriptional regulator	SP_1863	1.3	0.006
RNA methyltransferase	SP_1901	1.3	0.006
PDZ domain-containing protein	SP_1967	1.3	0.016
Thiamine diphosphokinase	SP_1982	1.3	>0.001
Ribulose-phosphate 3-epimerase	SP_1983	1.3	0.005
XRE family transcriptional regulator	SP_1989	1.3	0.027
Methyltransferase domain-containing protein	SP_2103	1.3	0.007
Hypothetical protein	SP_2187	1.3	0.005
DNA-binding response regulator	SP_2193	1.3	0.002
Rod shape-determining protein MreD	SP_2217	1.3	>0.001
Cell shape-determining protein MreC	SP_2218	1.3	0.012
Energy-coupling factor transporter transmembrane protein EcfT	SP_2219	1.3	0.009
Hypothetical protein	SP_0077	1.4	0.028
Phosphoglycolate phosphatase	SP_0104	1.4	0.001
Pullulanase	SP_0268	1.4	>0.001
Lanthionine synthetase	SP_0380	1.4	0.014
DNA-binding response regulator	SP_0387	1.4	0.002
Hypothetical protein	SP_0679	1.4	0.026
Energy coupling factor transporter S component ThiW	SP_0723	1.4	0.037
Hydroxyethylthiazole kinase	SP_0724	1.4	0.017
Foldase	SP_0981	1.4	0.002
Glucose-1-phosphate adenylyltransferase	SP_1122	1.4	0.005
Glucose-1-phosphate adenylyltransferase subunit GlgD	SP_1123	1.4	0.002
Glycogen synthase	SP_1124	1.4	>0.001
N-acetyltransferase	SP_1419	1.4	0.008
Class I SAM-dependent methyltransferase	SP_1578	1.4	0.004
Hypothetical protein	SP_1641	1.4	0.008

ABC transporter permease	SP_1652	1.4	0.008
Hypothetical protein	SP_1703	1.4	0.030
Type II toxin-antitoxin system HicB family antitoxin	SP_1786	1.4	>0.001
Hypothetical protein	SP_1810	1.4	0.005
Nicotinamide riboside transporter PnuC	SP_1859	1.4	0.030
Glycine/betaine ABC transporter permease	SP_1860	1.4	0.003
ABC transporter ATP-binding protein	SP_1861	1.4	0.004
DNA recombination protein RmuC	SP_1981	1.4	>0.001
Hypothetical protein	SP_2005	1.4	0.044
Sigma-70 family RNA polymerase sigma factor	SP_2006	1.4	0.014
MarR family transcriptional regulator	SP_2062	1.4	0.042
Gamma-glutamyl-gamma-aminobutyrate hydrolase family protein	SP_2072	1.4	0.026
YhgE/Pip domain-containing protein	SP_2233	1.4	0.030
Sigma-70 family RNA polymerase sigma factor	SP_0014	1.5	0.007
DUF4299 domain-containing protein	SP_0096	1.5	0.006
Hypothetical protein	SP_0117	1.5	0.001
DNA-binding response regulator	SP_0156	1.5	>0.001
Alcohol dehydrogenase AdhP	SP_0285	1.5	>0.001
Sensor histidine kinase	SP_0386	1.5	>0.001
Hypothetical protein	SP_0389	1.5	0.003
Hypothetical protein	SP_0449	1.5	0.005
Integrase	SP_0506	1.5	0.005
ATP-binding protein	SP_0570	1.5	0.001
Hypothetical protein	SP_0721	1.5	0.001
Hypothetical protein	SP_0826	1.5	0.002
DUF1919 domain-containing protein	SP_0907	1.5	0.005
GTP pyrophosphokinase	SP_1097	1.5	0.046
Glycogen-branching enzyme	SP_1121	1.5	>0.001
Immunoglobulin A1 protease	SP_1154	1.5	0.002
6-phospho-beta-galactosidase	SP_1184	1.5	0.054
Nicotinate phosphoribosyltransferase	SP_1421	1.5	>0.001
ABC transporter ATP-binding protein	SP_1580	1.5	>0.001
Hypothetical protein	SP_1635	1.5	0.022
N-acetyltransferase	SP_1807	1.5	0.002
Galactose-1-phosphate uridylyltransferase	SP_1829	1.5	0.006
ABC transporter ATP-binding protein	SP_2003	1.5	0.047
Type II/IV secretion system protein	SP_2053	1.5	0.010
Sensor histidine kinase	SP_2192	1.5	>0.001
ABC transporter ATP-binding protein	SP_0242	1.6	0.001
ECF transporter S component	SP_0488	1.6	>0.001
ABC transporter ATP-binding protein	SP_0707	1.6	0.002
Amino acid ABC transporter ATP-binding protein	SP_0709	1.6	0.002

Protein kinase	SP_1061	1.6	0.003
NAD (+) kinase	SP_1098	1.6	>0.001
Hypothetical protein	SP_1145	1.6	>0.001
Transcription antiterminator	SP_1187	1.6	0.005
ABC transporter ATP-binding protein	SP_1282	1.6	>0.001
Hypothetical protein	SP_1385	1.6	>0.001
NAD (+) synthetase	SP_1420	1.6	>0.001
Hypothetical protein	SP_1452	1.6	0.002
Hypothetical protein	SP_1679	1.6	0.001
Transposase	SP_1806	1.6	>0.001
UDP-glucose 4-epimerase GalE	SP_1867	1.6	>0.001
PTS ascorbate transporter subunit IIC	SP_2038	1.6	0.032
Membrane protein	SP_2132	1.6	>0.001
PTS ascorbate transporter subunit IIC	SP_2133	1.6	>0.001
Choline-binding protein A	SP_2190	1.6	>0.001
Endo-alpha-N-acetylgalactosaminidase	SP_0368	1.7	0.011
Transporter	SP_0385	1.7	>0.001
Transcription antiterminator	SP_0395	1.7	0.032
ISL3 family transposase	SP_0460	1.7	0.004
Peptidase M50 family protein	SP_0694	1.7	0.031
PspC domain-containing protein	SP_0910	1.7	>0.001
ABC transporter ATP-binding protein	SP_0957	1.7	0.002
Phosphate acetyltransferase	SP_1100	1.7	>0.001
Type II toxin-antitoxin system RelE/ParE family toxin	SP_1143	1.7	>0.001
1-alkyl-2-acetylglycerophosphocholine esterase	SP_1450	1.7	>0.001
Cof-type HAD-IIB family hydrolase	SP_1451	1.7	>0.001
Hypothetical protein	SP_1562	1.7	0.031
PRD domain-containing protein	SP_1621	1.7	0.001
DUF4162 domain-containing protein	SP_1717	1.7	>0.001
DUF2273 domain-containing protein	SP_1803	1.7	0.005
CsbD family protein	SP_1805	1.7	>0.001
Metal-sulfur cluster assembly factor	SP_2125	1.7	>0.001
Alpha-1%2C2-mannosidase	SP_2145	1.7	>0.001
Iron ABC transporter substrate-binding protein	SP_0243	1.8	0.001
Trans-2-decenoyl-[acyl-carrier protein] isomerase	SP_0415	1.8	0.027
Quorum-sensing system pheromone BlpC	SP_0528	1.8	0.011
RluA family pseudouridine synthase	SP_1099	1.8	>0.001
Lactose PTS system EIICB component	SP_1185	1.8	0.002
ROK family protein	SP_1324	1.8	0.022
Site-specific DNA-methyltransferase	SP_1431	1.8	>0.001
ROK family protein	SP_1675	1.8	>0.001
DUF624 domain-containing protein	SP_1677	1.8	0.001

LacI family transcriptional regulator	SP_1799	1.8	>0.001
Aspartate aminotransferase	SP_1800	1.8	0.001
Asp23/Gls24 family envelope stress response protein	SP_1804	1.8	>0.001
DUF1304 domain-containing protein	SP_2061	1.8	>0.001
Dihydroxy-acid dehydratase	SP_2126	1.8	>0.001
L-fucose isomerase	SP_2158	1.8	>0.001
D-alanine--poly(phosphoribitol) ligase	SP_2176	1.8	>0.001
Isoprenylcysteine carboxyl methyltransferase	SP_2191	1.8	>0.001
Ribosomal subunit interface protein	SP_2206	1.8	>0.001
ATP-dependent Clp protease ATP-binding subunit	SP_0338	1.9	>0.001
Pts system mannitol-specific eiicb component	SP_0394	1.9	>0.001
Hypothetical protein	SP_0430	1.9	0.002
CPBP family intramembrane metalloprotease	SP_0547	1.9	>0.001
Hypothetical protein	SP_0558	1.9	0.001
Hypothetical protein	SP_0696	1.9	0.008
Amino acid ABC transporter permease	SP_0710	1.9	>0.001
Hypothetical protein	SP_0956	1.9	>0.001
ABC transporter permease	SP_0958	1.9	0.000
PTS fructose transporter subunit IIB	SP_1197	1.9	0.008
V-type ATP synthase subunit D	SP_1315	1.9	0.021
V-type ATP synthase subunit C	SP_1319	1.9	0.003
Hypothetical protein	SP_1350	1.9	0.050
N-acetylneuraminate lyase	SP_1676	1.9	>0.001
YhcH/YjgK/YiaL family protein	SP_1680	1.9	>0.001
Carbohydrate ABC transporter permease	SP_1681	1.9	>0.001
ABC transporter ATP-binding protein	SP_1704	1.9	>0.001
Hypothetical protein	SP_1706	1.9	>0.001
Hypothetical protein	SP_1708	1.9	>0.001
Hypothetical protein	SP_2071	1.9	0.037
Hypothetical protein	SP_2122	1.9	>0.001
D-alanyl-lipoteichoic acid biosynthesis protein DltD	SP_2173	1.9	>0.001
D-alanine--poly(phosphoribitol) ligase subunit 2	SP_2174	1.9	>0.001
D-alanyl-lipoteichoic acid biosynthesis protein DltB	SP_2175	1.9	>0.001
